# Case Report of Rare, Extensive, and Resistant Pyoderma Gangrenosum Treated Successfully With GCSF

**DOI:** 10.1155/crdm/4265443

**Published:** 2025-10-01

**Authors:** Mahdi Ghahartars, Abolfazl Khalafi-Nezhad, Hossein Cheshmeh Ghasabani, Mojgan Akbarzadeh Jahromi

**Affiliations:** ^1^Department of Dermatology, Shiraz University of Medical Sciences, Shiraz, Iran; ^2^Department of Internal Medicine, Shiraz University of Medical Sciences, Shiraz, Iran; ^3^Department of Pathology, Shiraz University of Medical Sciences, Shiraz, Iran

**Keywords:** case report, granulocyte colony-stimulating factor, pyoderma gangrenosum

## Abstract

Pyoderma gangrenosum (PG) is a rare, noninfectious, autoimmune neutrophilic dermatosis comprising significant diagnostic and therapeutic challenges. It often presents as painful ulcerations and is frequently associated with systemic diseases such as inflammatory bowel disease and rheumatoid arthritis. Standard treatments, including corticosteroids, methotrexate, cyclosporine, and biologics like infliximab, can have variable efficacy, necessitating individualized approaches. This case report describes a 21-year-old Iranian male with extensive ulcerative plaques unresponsive to conventional treatments. The patient's history of severe anemia and familial incidence of PG suggested an underlying genetic predisposition. Remarkably, the patient showed significant improvement with granulocyte colony-stimulating factor (GCSF) therapy, which is not a standard treatment for PG. The successful use of GCSF in this case highlights its potential as an additive or alternative therapy for refractory PG, likely due to its ability to enhance neutrophil function, modulate immune responses, and promote wound healing. This case emphasizes the importance of personalized treatment strategies and the need for further research into novel therapeutic options for PG.

## 1. Introduction

Pyoderma gangrenosum (PG) is an uncommon, autoimmune neutrophilic dermatosis that encompasses great diagnostic and therapeutic challenges. It usually presents with painful ulcerations and predominantly affects middle-aged females [1, 2]. The classical ulcerative form of PG begins as small, tender, violaceous papules that rapidly evolve into deep ulcers with violaceous borders [1]. Despite its distinctive clinical features, PG remains a diagnosis of exclusion, often requiring histopathological examination to rule out other etiologies. PG is often associated with systemic disorders including inflammatory bowel disease (IBD), rheumatoid arthritis, hematological malignancies, and vasculitis which complicate its management [1–3]. Treatment selection in PG is frequently chosen by comorbidities; in patients with IBD, therapies that address both disorders (e.g., antitumor necrosis factor agents such as adalimumab) are often preferred [4].

Standard treatments comprise immunosuppressive agents such as corticosteroids, methotrexate, cyclosporine, and biologic medications like infliximab [5]. Still, treatment responses differ widely, and some patients do not respond to conventional medication. For refractory disease, therapeutic choices expand from corticosteroids and calcineurin inhibitors to include targets and adjuncts such as intravenous immunoglobulin (IVIG) [5]. Granulocyte colony-stimulating factor (GCSF) is mainly recognized for its ability to stimulate the bone marrow to generate granulocytes and stem cells, which are subsequently released into the bloodstream. While not a standard treatment for PG, its use is backed by several case reports and studies indicating its effectiveness in cases where conventional treatments have been unsuccessful [6]. The precise mechanism through which GCSF alleviates PG symptoms remains unclear, but it is hypothesized that GCSF may enhance neutrophil function and boost immune responses, thereby promoting ulcer healing. Therefore, individualized therapeutic approaches become preferable with respect to each case.

This case report presents a 21-year-old Iranian man with refractory PG, highlighting the potential role of GCSF as an alternative therapy. Despite conventional treatments failing, the patient showed significant improvement with GCSF [6].

## 2. Case Presentation

**Figure 1:**
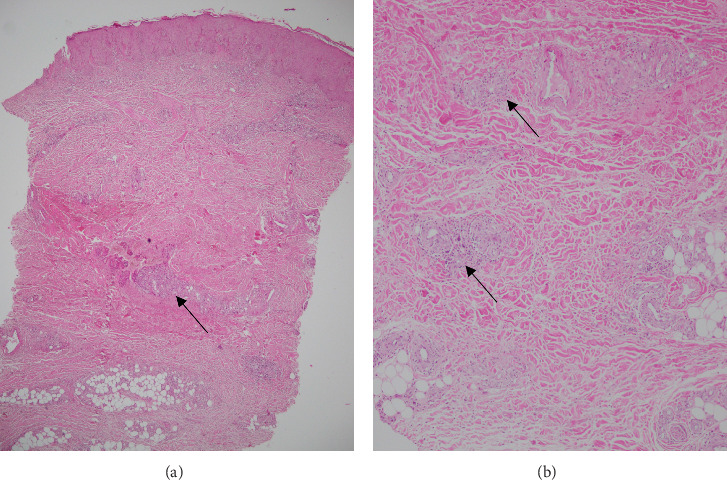
Superficial and deep perivascular mixed infiltration of lymphocytes, histiocytes, and polymorphonuclear leukocytes (PMNs), with associated dermal fibrosis (black arrows). (a) Hematoxylin and eosin (H&E), × 40; (b) H&E, × 200.

**Figure 2:**
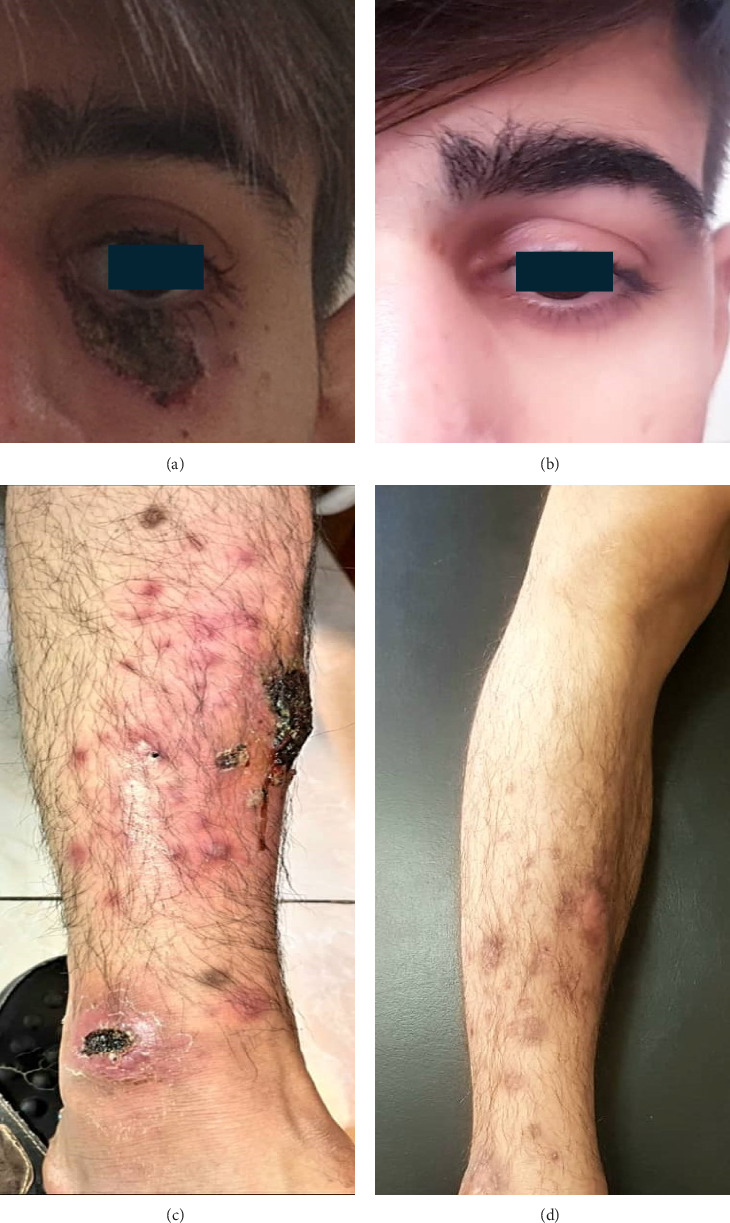
Clinical improvement in patient's ulcerative lesions after administration of granulocyte colony-stimulating factor ((a, c) ulcers during conventional treatment; (b, d) improvements in lesions 2 weeks after granulocyte colony-stimulating factor administration).

A 21-year-old Iranian male presented to our hospital with a 12-month history of extensive ulcerated plaques all over his body. The patient developed severe anemia at the age of 13 with no evidence of cellular proliferation disorder or storage disease which resolved spontaneously. Furthermore, he had a history of nodulocystic acne with a severe infection when he was 17 which were treated accordingly. The patient's brother was diagnosed with PG at the age of 35. These new painful plaques started from below his left orbit as acne-like lesions that spread to his upper extremity, abdomen, genitalia, and lower extremity with no amelioration. The skin punch biopsy was performed which showed hyperkeratosis, acanthosis, mild spongiosis, focal loss of granular layer, and exocytosis of some lymphocytes in the epidermis. Perivascular lymphocytic, eosinophilic, and polymorphonuclear infiltration, increased vascularity, RBC extravasation, hemosiderin-laden macrophages, and mild dermal fibrosis were seen in the dermis layer (Figure 1). These histopathological findings and ruling out infectious disorders were consistent with the diagnosis of PG. After that, he received methotrexate 10 mg weekly, cyclosporine 100 mg twice a day, and infliximab 400 mg monthly from the beginning of the disease course up to 1 year which resulted in no clinical improvement. His laboratory results revealed two consecutive neutropenia in the course of treatment. Accordingly, GCSF was prescribed for the patient in a weekly manner. The patient's symptoms started to improve just a few days after his second GCSF injection, and the ulcers disappeared completely after 14 days (Figure 2). Eventually, the patient has been evaluated every 3 months, at his follow-up visits.

## 3. Discussion

Herein, we presented a patient with refractory PG treated successfully with GCSF. The case presented here reveals the diagnostic and therapeutic challenges caused by PG, especially in young patients. The patient was presented with extensive ulcerative plaques that did not respond to conventional treatments, including methotrexate, cyclosporine, and infliximab. This resistance to routine medications is not uncommon in PG and highlights the need for other treatment strategies [5, 6]. Furthermore, the patient's history of severe anemia and his familial history suggest a possible underlying systemic or genetic predisposition. Although no overt systemic disease was identified in this patient, the family history of PG points to a potential genetic link that needs to be further investigated; still, the patient did not consent to a genetic study in this regard. Genetic studies, such as those exploring mutations in genes like PSTPIP1, have shown promise in understanding the hereditary aspects of PG [7]. The successful use of GCSF in this case shows a promising medication for refractory PG. However, it needs future research, especially in the treatment of patients concerning different gene mutations, to validate these findings.

In refractory PG, several strategies have been supported by literature [5]. Escalation or combination systemic immunosuppression (high-dose corticosteroids with cyclosporine) remains a common step; biologic therapies are increasingly used early, particularly when IBD or arthritis co-exists, including anti-TNF agents (infliximab, adalimumab), IL-1 blockade (anakinra/canakinumab), IL-12/23 inhibition (ustekinumab), IL-17/23–axis agents, and JAK inhibitors (e.g., tofacitinib) [1]. IVIG is an adjunct option with reported benefit in treatment-resistant cases [8, 9].

GCSF is primarily known for its role in stimulating the bone marrow to produce granulocytes and stem cells, which are then released into the bloodstream. Its use in PG, while not standard, is supported by a few case reports and studies suggesting its efficacy in cases where conventional treatments have failed. The exact mechanism by which GCSF improves PG symptoms is not well understood, but it is hypothesized that GCSF may enhance neutrophil function and improve host immune responses, thereby aiding in the resolution of the ulcers [1, 2, 6]. The improvement observed with GCSF treatment in this case is notable. Similarly, a study by Jabbari et al. described a patient with PG and cyclic neutropenia who responded well to GCSF, leading to rapid ulcer healing [6]. However, there are some reports of PG attributed to GCSF administration [10, 11]. Mizushima et al. report a patient with myelodysplastic syndrome-related pancytopenia who received GCSF and developed ulcers diagnosed as PG [12].

The mechanism by which GCSF aids in the treatment of PG involves multiple hypothesized pathways. GCSF enhances neutrophil function by stimulating their production and maturation, improving their capacity to combat inflammation [13]. It also has immunomodulatory effects, influencing other immune cells such as macrophages and T-cells to regulate the immune response [14]. Additionally, GCSF exerts direct anti-inflammatory effects, promoting the resolution of excessive inflammation characteristic of PG [13, 15]. Its role in promoting angiogenesis and wound healing is also significant, as it improves blood supply to affected areas, facilitating better tissue repair [16, 17]. In patients with neutropenia, GCSF corrects this condition, reducing infection risks and allowing better inflammation management [18]. These combined effects make GCSF a promising option for treating refractory PG, as demonstrated in the presented case where conventional treatments failed. Although GCSF demonstrated rapid clinical benefit in our patient, its use is not without risks. Hypersensitivity to GCSF preparations or their excipients is considered an absolute contraindication. Relative concerns include patients with myeloid malignancies, where it may theoretically worsen these comorbidities. In addition, paradoxical cases of GCSF-induced PG have been described, particularly in patients with underlying hematologic disorders or during pregnancy [8–10]. Therefore, careful patient selection and close monitoring are required when considering GCSF as an adjunctive treatment for refractory PG. However, we suggest that future clinical trials with larger sample sizes should be implemented to validate our findings.

In conclusion, this case highlights the importance of considering alternative therapies such as GCSF for treating refractory PG. It also emphasizes the need for a personalized approach in managing PG, given the variability in treatment response. Further research into the mechanisms of GCSF and its role in PG, genetic studies to understand familial patterns, and clinical trials to assess its efficacy could provide valuable insights into more effective management strategies for this challenging condition.

## References

[1] Dissemond J., Marzano A. V., Hampton P. J., Ortega-Loayza A. G. (2023). Pyoderma Gangrenosum: Treatment Options. *Drugs*.

[2] Brooklyn T., Dunnill G., Probert C. (2006). Diagnosis and Treatment of Pyoderma Gangrenosum. *BMJ*.

[3] Ye M. J., Ye J. M. (2014). Pyoderma Gangrenosum: A Review of Clinical Features and Outcomes of 23 Cases Requiring Inpatient Management. *Dermatology Research and Practice*.

[4] Fousekis F. S., Mpakogiannis K., Karampinis E. (2025). Pyoderma Gangrenosum in a Patient With Crohn’s Disease Treated With Adalimumab: A Case-Based Review and Systematic Review of the Current Literature. *Clinical Practice*.

[5] Maronese C. A., Pimentel M. A., Li M. M., Genovese G., Ortega-Loayza A. G., Marzano A. V. (2022). Pyoderma Gangrenosum: An Updated Literature Review on Established and Emerging Pharmacological Treatments. *American Journal of Clinical Dermatology*.

[6] Jabbari H., Payvarmehr F., SeyedAlinaghi S., Roosta N. (2011). A Case Report of Cyclic Neutropenia Associated With Pyoderma Gangrenosum. *Acta Medica Iranica*.

[7] Shoham N. G., Centola M., Mansfield E. (2003). Pyrin Binds the PSTPIP1/CD2BP1 Protein, Defining Familial Mediterranean Fever and PAPA Syndrome as Disorders in the Same Pathway. *Proceedings of the National Academy of Sciences of the U S A*.

[8] McKray-Smith K., Schroeder Q., Curlis K. (2025). A Decade-Long Case of Pyoderma Gangrenosum Successfully Treated With a Targeted Multimodal Protocol: A Case Report. *Foot and Ankle Surgery: Techniques, Reports & Cases*.

[9] Song H., Lahood N., Mostaghimi A. (2018). Intravenous Immunoglobulin as Adjunct Therapy for Refractory Pyoderma Gangrenosum: Systematic Review of Cases and Case Series. *British Journal of Dermatology*.

[10] Lewerin C., Mobacken H., Nilsson-Ehle H., Swolin B. (1997). Bullous Pyoderma Gangrenosum in a Patient with Myelodysplastic Syndrome during Granulocyte Colony-Stimulating Factor Therapy. *Leukemia and Lymphoma*.

[11] Weiß K. T., Berneburg M., Santjohanser C., Karrer S. (2017). Pyoderma Gangrenosum on G-CSF (Lenograstim) Treatment During Pregnancy. *JDDG: Journal der Deutschen Dermatologischen Gesellschaft*.

[12] Mizushima M., Miyoshi H., Yonemori K. (2021). Pyoderma Gangrenosum After Total Hip Arthroplasty Associated With Administration of Granulocyte Colony-Stimulating Factor: A Case Report. *JBJS Case Connector*.

[13] Panopoulos A. D., Watowich S. S. (2008). Granulocyte Colony-Stimulating Factor: Molecular Mechanisms of Action During Steady State and ‘Emergency’ Hematopoiesis. *Cytokine*.

[14] Bhattacharya P., Thiruppathi M., Elshabrawy H. A., Alharshawi K., Kumar P., Prabhakar B. S. (2015). GM-CSF: An Immune Modulatory Cytokine That Can Suppress Autoimmunity. *Cytokine*.

[15] Yousefi S., Simon D., Stojkov D., Karsonova A., Karaulov A., Simon H. U. (2020). In Vivo Evidence for Extracellular DNA Trap Formation. *Cell Death & Disease*.

[16] Huang H., Zhang Q., Liu J., Hao H., Jiang C., Han W. (2017). Granulocyte-Colony Stimulating Factor (G-CSF) Accelerates Wound Healing in Hemorrhagic Shock Rats by Enhancing Angiogenesis and Attenuating Apoptosis. *Medical Science Monitor*.

[17] Everts P. A., Lana J. F., Onishi K. (2023). Angiogenesis and Tissue Repair Depend on Platelet Dosing and Bioformulation Strategies Following Orthobiological Platelet-Rich Plasma Procedures: A Narrative Review. *Biomedicines*.

[18] Căinap C., Cetean-Gheorghe S., Pop L. A. (2021). Continuous Intravenous Administration of Granulocyte-Colony-Stimulating Factors-A Breakthrough in the Treatment of Cancer Patients with Febrile Neutropenia. *Medicina*.

